# Evidence Supporting the Hydrophobic-Mismatch Model for Cytochrome *b*_6_*f*-Driven State Transitions in the Cyanobacterium *Synechocystis* Species PCC 6803

**DOI:** 10.3390/membranes15120383

**Published:** 2025-12-17

**Authors:** Terezia Kovacs, Laszlo Kovacs, Mihaly Kis, Michito Tsuyama, Sindhujaa Vajravel, Eva Herman, Nia Petrova, Anelia Dobrikova, Tomas Zakar, Svetla Todinova, Sashka Krumova, Zoltan Gombos, Radka Vladkova

**Affiliations:** 1 Synthetic and Systems Biology Unit, Institute of Biochemistry, HUN-REN Biological Research Centre, H-6726 Szeged, Hungary; 2 HCEMM-BRC Pharmacodynamic Drug Interaction Research Group, Hungarian Centre of Excellence for Molecular Medicine, H-6728 Szeged, Hungary; 3 Institute of Plant Biology, HUN-REN Biological Research Centre, H-6726 Szeged, Hungary; 4 Department of Agriculture, Forest and Forest Products Sciences, Plant Metabolic Physiology, Kyushu University, Fukuoka 812-8581, Japan; 5 Temasek Life Sciences Laboratory, National University of Singapore, Singapore 117604, Singapore; 6 Institute of Biophysics and Biomedical Engineering, Bulgarian Academy of Sciences, 1113 Sofia, Bulgaria; 7 Institute of Photonics and Electronics of the Czech Academy of Sciences, 18200 Prague, Czech Republic

**Keywords:** cytochrome *b*_6_*f*, circular dichroism, differential scanning calorimetry, hydrophobic mismatch, photosystem, PAL mutant, phycobilisome, State transitions, single-point mutation, *stn7 Arabidopsis* mutant

## Abstract

While there is a consensus that the cytochrome *b*_6_*f* complex (cyt*b*_6_*f*) in algae and plants is involved in the regulatory mechanism of oxygenic photosynthesis known as light-induced state transitions (STs), no such consensus exists for cyanobacteria. Here, we provide the first direct functional evidence for cyt*b*_6_*f* using single-point mutation data. We introduced a PetD-Phe124Ala substitution in the cyanobacterium *Synechocystis* sp. PCC 6803 to test the key predictions of the hydrophobic-mismatch (HMM) model for cyt*b*_6_*f*-driven STs in all oxygenic photosynthetic species. These predictions concern the role of the Phe/Tyr124*^fg^*^-loop-PetD^ and the extent and kinetic characteristics of STs. The effects of PetD-F124A mutation on STs were monitored using 77K and Pulse-Amplitude-Modulated (PAM) fluorescence. For comparison, we employed a phycobilisome (PBS)-less *Synechocystis* mutant and wild-type (WT) strain, as well as the *stn7* mutant and WT of *Arabidopsis* plant. The PetD-F124A mutation reduced the extent of STs and selectively affected the two-exponential kinetics components of the transitions. Under State 1 conditions, the mutant exhibited ~60% less energetic decoupling of PBS from photosystem I (PSI) compared to the WT. It is explainable by the HMM model with the inability of the PetD-F124A mutant, during the induction phase of the State 2→State 1 transition to adopt the cyt*b*_6_*f* conformation with minimal hydrophobic thickness. PAM-derived parameters indicated that PSII electron transport function is not inhibited, and no detectable effect on cyclic electron transport around PSI was observed under low-light conditions. Circular dichroism and differential scanning calorimetry confirmed that both the PSI trimer/monomer ratio and the structural integrity of the PBSs are preserved in the mutant. The compensatory response to the mutation includes decreased PSI content and an increase in PBS rod size. In conclusion, (1) cyt*b*_6_*f* is involved in cyanobacterial STs; (2) evidence is provided supporting the HMM model; (3) the electron transfer and signal transduction functions of cyt*b*_6_*f* are separated into distinct domains; and (4) the signaling pathway regulating STs and pigment-protein composition in *Synechocystis* involves PetD-Phe124.

## 1. Introduction

State transitions are a rapid (15–20 min) regulatory mechanism of oxygenic photosynthesis that is evolutionarily conserved from prokaryotic cyanobacteria to eukaryotic algae and plants [1,2,3,4,5]. Like the primary light reactions of oxygenic photosynthesis—light-harvesting, excitation energy transfer, charge separation, proton, and electron transfer—this process occurs in the thylakoid membranes of cyanobacterial cells and chloroplasts of algae and plants (e.g., [6]). State transitions represent a low-intensity light-controlled redistribution of excitation energy between photosystem II (PSII) and photosystem I (PSI) that optimizes photosynthetic efficiency [7]. In both cyanobacteria and chloroplasts, State transitions are activated by the gradual reduction or oxidation of the plastoquinol (PQ) pool in response to changes in light quality. State 1 is established upon preferential excitation of PSI (e.g., by far-red light). State 2 is achieved upon preferential excitation of PSII (by red light) (see, e.g., [8] for a recent comprehensive survey). The State transition process comprises an induction phase, onset, and progression to completion [9]. Although the induction phases of State transitions and the final outcome of State transitions are very similar in cyanobacteria and chloroplasts [8,9], they differ in the activation of light-harvesting complex II (LHCII) kinase/phosphatase pairs in chloroplasts [10,11,12,13], which are missing in cyanobacteria [14,15]. While the role of cyt*b*_6_*f* in activating LHCII kinases in chloroplasts is well established [16,17,18,19], its involvement in cyanobacterial State transitions remains controversial [8,14,15,20,21]. On the one hand, Calzadilla et al. (2019) [15] used chemical inhibitors and concluded that cyt*b*_6_*f* is not involved in cyanobacterial State transitions. On the other hand, recent findings by Wei et al. (2025) [22], using a *petN* mutant (lacking the PetN small subunit of cyt*b*_6_*f*), strongly suggest that cyt*b*_6_*f* is required for State transitions in cyanobacteria. Clarifying the role of cyt*b*_6_*f* in cyanobacterial State transitions is crucial for understanding the evolution and diversity of photosynthetic regulation.

**Figure 1:**
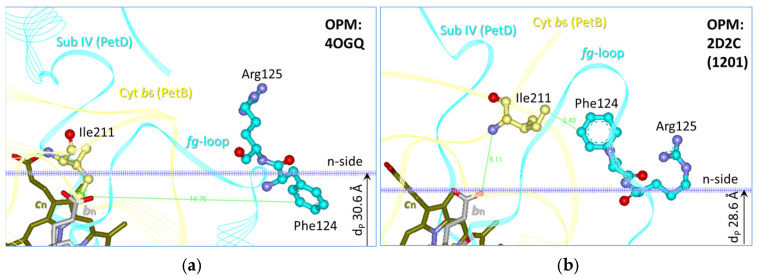
Flexibility center in cyt*b*_6_*f*, the amino acid Phe124*^fg^*^-loop-PetD^, which is substituted by Ala in the present study. The *fg*-loop region is shown for two cyt*b*_6_*f* conformations, corresponding to the induction phase of the transition to State 1, during which the hydrophobic thickness of cyt*b*_6_*f* changes from 30.6 Å (**a**) to 28.6 Å (**b**) [8,9]. (**a**) Structure corresponding to the optimal oxidized state of the PQ pool under low-light conditions, and (**b**) structure corresponding to the over-oxidized state of the PQ pool. The depicted structures, including the n-side interface and cyt*b*_6_*f* hydrophobic thickness, are taken from the OPM database [24]. Note that the Phe*^fg^*^-loop-PetD^ is evolutionarily conserved in cyanobacteria, plants, and some algae, whereas Tyr*^fg^*^-loop-PetD^ is found in only ten algal species [9]. The adjacent residue, Arg125*^fg^*^-loop-PetD^, is discussed in detail in [25,26].

A unified hydrophobic mismatch (HMM) model for cyt*b*_6_*f*-driven State transitions across all oxygenic photosynthetic organisms was proposed by Vladkova in 2015 [9]. This biophysical model is based on the concept of HMM-induced lipid sorting around a membrane protein [23]. HMM arises when the hydrophobic thickness of the protein (d_P_) differs from that of the surrounding lipid bilayer (d_L_). This energetically unfavorable condition generates a driving force that relaxes the mismatch through lipid reorganization. Specifically, lipid sorting around cyt*b*_6_*f*—and concomitant lipid depletion at other membrane sites—triggers structural reorganization of antenna-PSs super- and megacomplexes. Key molecular players in this model include Chlorophyll *a* (Chl*a*), several amino acid residues, and two types of lipid sorting around cyt*b*_6_*f*, depending on the sign of cyt*b*_6_*f*-induced HMM. Notably, the Phe/Tyr124*^fg^*^-loop-PetD^ residue in the *fg*-loop of the PetD subunit—located on the cytoplasmic/stromal (n-side) of cyt*b*_6_*f* (see Figure 1)—has been identified as critical for State transitions in both cyanobacteria and chloroplasts [9]. The aromatic ring at this position is evolutionarily conserved: it is Phe in cyanobacteria, plants, and some algae, and Tyr in ten algal species [9]. Very recently, the HMM model has been extended and augmented with identifying the two types of lipid sorting around cyt*b*_6_*f*: monogalactosyldiacylglycerol (MGDG) sorts around cyt*b*_6_*f* to transiently compensate for the cyt*b*_6_*f*-induced positive HMM (d_P_ > d_L_) during the induction phase of the transition to State 2, when the PQ pool becomes progressively reduced, and digalactosyldiacylglycerol (DGDG) sorts around cyt*b*_6_*f* to transiently compensate for the cyt*b*_6_*f*-induced negative HMM (d_P_ < d_L_) during the induction phase of the transition to State 1, when the PQ pool becomes progressively oxidized. This updated model clarifies that lipids—not proteins—serve as the primary effectors of signals transmitted from cyt*b*_6_*f* to light-harvesting antenna–PSs super- and megacomplexes during State transitions. Moreover, the HMM model identifies cyt*b*_6_*f* as the first photosynthetic protein known to actively exploit the evolutionarily conserved, unique four-lipid-class composition of thylakoid membranes [8]. It also provides a detailed spatiotemporal sequence of molecular events—from induction through onset to completion—of State transitions in both cyanobacteria and chloroplasts. In cyanobacteria, signal transduction is purely biophysical: cyt*b*_6_*f* triggers lipid reorganization, which in turn drives phycobilisome (PBS)-PSs reorganization. In chloroplasts, signal transduction is combined biophysical and biochemical: lipid reorganization facilitates kinase recruitment around and activation by cyt*b*_6_*f*, leading to LHCII phosphorylation and p-LHCII-PSs reorganization.

Meanwhile, a comparative study of the wild-type (WT) alga *Chlamydomonas reinchardtii* (hereafter *Chlamydomonas*) and its cyt*b*_6_*f* mutants [25] has confirmed key predictions of the HMM model [9] regarding Phe/Tyr124: (1) Tyr124*^fg^*^-loop-PetD^ is important for algal State transitions, (2) Phe and Tyr are functionally equivalent at position 124 of PetD, (3) Tyr124*^fg^*^-loop-PetD^ is not involved in the electron-transfer function of cyt*b*_6_*f*, and (4) the proposed kinase-binding role of Phe/Tyr124 [9] has not been confirmed in vivo. However, in vitro experiments showed that both Tyr124 and Arg125 enhance the autophosphorylation of the n-side Stt7 kinase domain, and that Arg125 is directly involved in this process (Tyr124 was not tested for this specific role in [25]). While these confirmations primarily relate to the role of Phe/Tyr124*^fg^*^-loop-PetD^ during the transition to State 2—a process mediated by LHCII kinase in algae and plants [10,11]—the study by [25] did not address which cyt*b*_6_*f* residues, if any, are important for the transition to State 1.

On the other side, the HMM model [8,9] also assigns a critical role of Phe/Tyr124 during the transition to State 1. According to this model, in both cyanobacteria and chloroplasts, this residue constitutes a flexibility center at the cytoplasmic/stroma (n-) side of cyt*b*_6_*f* (see Figure 1) and is part of signaling platforms that originate at the Chl*a* molecule and propagate to Phe/Tyr124, mediated by several amino acids [9] and a network of lipids and the carotenoid [8]. The transition to State 1 involves significant conformational changes in the *fg*-loop region (Figure 1). Specifically, the detachments of sulfoquinovosyldiacylglycerol (SQDG) and phosphatidylglycerol (PG) molecules and the binding of two DGDG molecules to cyt*b*_6_*f*, is accompanied by the progressive movement and rotation of the aromatic ring of Phe124*^fg^*^-loop-PetD^—from a membrane-buried position (Figure 1a) toward the n-side hydrophilic region, approaching Ile211^cyt*b*6^ and Leu106^cyt*b*6^ (Figure 1b, see [8], Suppl_Images-2.pdf for further details).

Cyanobacteria (prokaryotes) differ from algae and plants (eukaryotes) in both their light-harvesting antenna complexes and thylakoid architecture. In cyanobacteria, the principal antenna for PSII is the giant water-soluble PBS [27,28], which can also serve as an antenna for PSI [29,30]. In contrast, chloroplasts utilize integral-membrane antenna complexes—LHCII for PSII and LHCI for PSI [31,32]. Moreover, plants and green algal chloroplasts feature a complex 3D architecture composed of stacked (grana) and unstacked (stromal) regions (e.g., [33]), whereas cyanobacteria lack grana regions (e.g., [34]). As noted above, cyanobacterial State transitions differ from those in chloroplasts due to the absence of kinase/phosphatase pair mediators. It has been proposed that these kinase/phosphatase pairs are involved in the State transitions of algae and plants, facilitating the reorganization of antenna-PSs [9].

Confirming the HMM model in cyanobacteria would provide significant new insights for several reasons: (1) It would identify Chl*a* within cyt*b*_6_*f* as the crucial redox sensor and transmembrane signal transmitter, clarifying why Chl*a* and cyt*b*_6_*f* form an evolutionarily conserved functional pair [9]; (2) It would establish Phe124*^fg^*^-loop-PetD^ as the most critical amino acid residue in cyt*b*_6_*f* for State transitions [9], opening avenues for targeted manipulation to enhance photosynthetic productivity and enable biotechnological applications; (3) It would reveal the driving force behind the structural reorganization of antenna-PSs super- and megacomplexes, explaining why the unique four-lipid-class composition of thylakoid membranes has been conserved alongside cyt*b*_6_*f* throughout evolution [8]—and thereby enabling rational engineering of lipid composition; (4) Given that cyanobacteria are the evolutionary ancestors of chloroplasts, such confirmation would not only elucidate the origin and diversification of this ancient regulatory mechanism during the evolution of oxygenic photosynthesis but would also demonstrate that State transitions represent a purely biophysical regulatory process that predates the later evolutionary addition of kinase/phosphatase pairs in eukaryotes.

Since the study by Calzadilla et al. [15] lacks direct evidence that the same cyt*b*_6_*f* mutations known to affect algal state transitions [25,35] have no effect in cyanobacteria, substituting Phe124 with a very small, nonpolar residue such as Ala, represents the most direct experimental approach to concomitantly address two key questions: first, whether cyt*b*_6_*f* is involved in cyanobacterial State transitions, and second, whether the HMM model applies in vivo to cyanobacteria. To achieve this goal, in the present work, we substituted Phe124 with Ala by site-directed mutagenesis in the cyanobacterium *Synechocystis* sp. PCC 6803 (hereafter *Synechocystis*). Our data strongly indicate that cyt*b*_6_*f* serves as the central hub for State transitions regulation and signaling in cyanobacteria—as it does in algae and plants—and that the HMM model is valid in vivo for cyanobacterial State transitions. Furthermore, we employed the PAL mutant (lacks PBSs) of *Synechocystis* and both the WT (Col-0) and *stn7* mutant of *Arabidopsis thaliana* (hereafter *Arabidopsis*) to compare the effects of missing light-harvesting antenna and the absence of LHCII kinase on State transitions kinetics with those observed in the PetD-F124A mutant. These comparisons provide additional support for the validity of the HMM model in plants as well. This study reveals that the Phe124*^fg^*^-loop-PetD^ probably participates in a signaling pathway that regulates PSI content and PBS rod size.

## 2. Materials and Methods

### 2.1. Cyanobacterial Cell Growth Conditions, Growth Curves, and Plant Material

*Synechocystis* sp. PCC 6803 WT, PetD-F124A mutant (generated in this study, available in GenomeNet database, accession number T00004), and the PAL mutant [36] were cultivated in BG11 medium [37] supplemented with 5 mM HEPES–NaOH (pH 7.5). Cultures were grown photoautotrophically in 100 mL or 250 mL Erlenmeyer flasks containing 50 mL or 100 mL of cell suspension, respectively, on a rotary shaker (120 rpm) at 30 °C. The cells were illuminated with continuous white fluorescent light at a photon flux density of 35 μmol photons m^−2^ s^−1^. Antibiotics were added to the medium: 40 μg mL^−1^ kanamycin for PetD-F124A and 7.5 μg mL^−1^ chloramphenicol for the PAL mutant.

Growth curves of *Synechocystis* WT and PetD-F124A strains were monitored by measuring optical density at 750 nm (OD750) every 24 h over 10 days. Three independent biological samples with an equal cell density (OD750 = 0.17 on day 0), were grown photoautotrophically in 1 L flasks containing 400 mL culture. Four-day cultures were used for the measurements.

*Arabidopsis thaliana* WT (ecotype Columbia, Col-0) and its *stn*7 mutant (SALK_073254, At1g68830) [11] were used. The *stn*7 is a T-DNA insertion mutant in the Col-0 background. The *stn*7 gene lacks the serine/threonine kinase STN7 required for the phosphorylation of LHCII and thus for State transition. Plants were grown in a growth chamber (60 µmol photons m^−2^ s^−1^, 8 h light/16 h dark illumination regime, 23 °C, 50–70% relative humidity) for 8–11 weeks.

### 2.2. Construction and Verification of Synechocystis PetD-Phe124Ala Point Mutant

The *petD* gene with flanking sequences was amplified by PCR using Q5^®^ High-Fidelity DNA Polymerase (New England Biolabs, Ipswich, MA, USA), genomic DNA template isolated from WT *Synechocystis*, and the primers cytb_6__for1 (5′AATCGTTTCCGGTGTACC-3′) and cytb_6__rev1 (5′-GCG**AAGCTT**TTAGAACAAGCCCAAGG-3′), and cytb_6__for2 (5′-GCG**AAGCTT**TGAAGATTTTCCACTCTTG-3′) and cytb_6__rev2 (5′-GCCGTATTGTTAGAAACC-3′).

**Figure 2:**
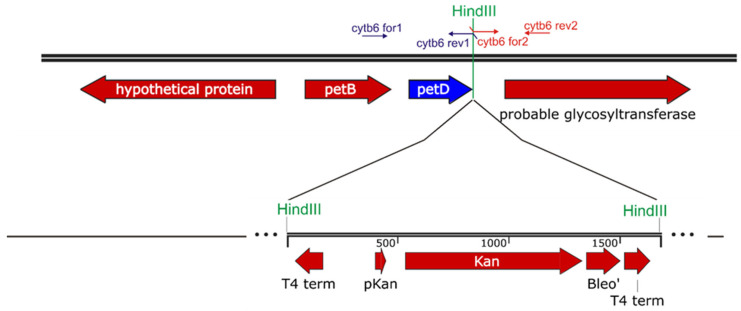
Physical map of the *Synechocystis* genome fragment containing the mutated *petD* gene. The *petD* gene with flanking sequences was amplified by PCR. A kanamycin cassette was cloned into the engineered *HindIII* site immediately after the coding region. Arrows indicate the position of the PCR primers.

The fragments were digested with *HindIII* restriction enzyme and cloned between *EcoRV* and *HincII* sites of the plasmid pBluescript II KS+. Thus, a *HindIII* site (bold) was engineered immediately downstream of the *petD* coding region. The sequence TTC, which codes for Phe124 of the PetD protein, was changed to GCA, which codes for Ala, using the Q5^®^ Site-Directed Mutagenesis Kit (New England Biolabs), resulting in PetD-F124A. Then, a kanamycin resistance gene was inserted into the *HindIII* site. This construct was used to transform *Synechocystis* cells (Figure 2). Transformants were selected on BG11 agar plates supplemented with glucose and increasing amounts of kanamycin by several rounds of single-colony restreaking. Complete segregation of the mutant cells was confirmed by PCR using primers cytb_6__for1 and cytb_6__rev2.

**Figure 3:**
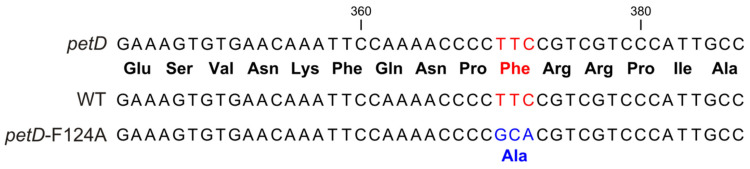
Comparison of the part of the *petD* sequence containing the mutated region, sequenced from the genomes of WT and PetD-F124A strains.

To verify the changes in the genome, a fragment containing the mutation site was amplified by PCR and sequenced. As seen in Figure 3, the mutant strain contains the expected GCA instead of WT TTC.

### 2.3. Absorption Spectroscopy and Determination of Pigment Content

Absorption spectra (400–800 nm) of intact cell suspensions at room temperature (~25 °C) were recorded using SPECORD 210 PLUS, Edition2010 spectrophotometer (Analytik-Jena AG, Jena, Germany) with a slit width of 0.5 nm and a scan speed of 1 nm/s. Chl*a* and phycocyanin (PC) contents were estimated from the whole-cell absorption spectra as described in [38]. Cell density was determined by measuring OD750 using both a SPECORD 210 PLUS and a Shimadzu UV-1601 spectrophotometer (Shimadzu Co., Kyoto, Japan). Pigment concentration, divided by the OD750 of the cell suspension, was used to measure the pigment content per cell. Chl*a* concentration was additionally determined spectrophotometrically after extraction in 90% (*v*/*v*) methanol, using the extinction coefficient given in [39].

### 2.4. Fluorescence Spectroscopy

Fluorescence emission spectra (610–800 nm) of intact cells upon Chl*a* (436 nm) and PBS (590 nm) excitation at 30 °C were recorded using a spectrofluorometer (Fluorolog-3/Jobin–Yvon–Spex Instrument S.A., Inc., Longjumeau, France) equipped with a thermostatted four-cuvette holder connected to a thermostat. The cell suspensions in 1 cm cuvettes had equal Chl*a* concentration of 2 μg mL^−1^. Before measurement, the samples were dark-adapted for 15 min in the cuvette holder at 30 °C. The slit-widths were 3 nm, the integration time was 1 s, and the increments were 0.5 nm.

Low-temperature (77K) fluorescence emission spectra (615–800 nm) were measured using the same spectrofluorometer equipped with a liquid-nitrogen cryostat. For these measurements, cells containing 3 μg of Chl*a* were filtered onto Whatman GF/C filter paper disk (25 mm diameter) (Whatman, Cytiva, Waltham, MA, USA) dark-adapted for 15 min, and immediately frozen in liquid nitrogen. To induce State 1, dark-adapted filter disks were illuminated for 16 min with a combination of red (635 nm, 8 µmol photons m^−2^ s^−1^) and far-red (720 nm, 6 µmol photons m^−2^ s^−1^) lights using the Dual-PAM fluorometer (Heinz Walz GmbH, Effeltrich, Germany), then immediately frozen. For State 2, disks were first treated the same as State 1 and then exposed to an additional 16 min red light alone, followed by immediate freezing. Fluorescence was excited at 590 nm, then at 436 nm on the same sample without changing its orientation. The slit-widths were 3 nm, the integration time was 1 s, and the increments were 0.5 nm. The spectra were corrected for photomultiplier sensitivity and light drifts.

### 2.5. Circular Dichroism Spectroscopy

Circular dichroism (CD) spectra were recorded between 350 and 800 nm at room temperature (~25 °C) using a Jasco J-815 dichrograph (Jasco, Tokyo, Japan). The instrument was set to a 5 nm bandpass and 1 nm resolution, with a scanning rate of 100 nm/min and a 4 s integration time. The Chl*a* content of the samples was adjusted to 15 µg mL^−1^ and measured in a 1 cm quartz cuvette. CD spectra were normalized to the Chl*a* red absorption maximum.

### 2.6. Differential Scanning Calorimetry

For differential scanning calorimetry (DSC) experiments, cyanobacterial cells (OD750 ~0.8–1.3) were harvested by centrifugation at 3900× *g* for 10 min at 10 °C and gently resuspended in BG-11 medium to a final concentration of 2–5 mg protein mL^−1^. Thermal scanning was performed from 25 to 120 °C with a heating rate of 1 °C min^−1^ using a DASM-4 differential scanning calorimeter (IBBP-RAS, Pushchino, Russia). Prior to measurement, samples were equilibrated at 25 °C for 20 min. A baseline thermogram (BG-11 medium vs. BG-11 medium) was recorded and subtracted from the sample scans. Resulting DSC profiles were normalized to total protein content, determined according to [40] and adapted for *Synechocystis* as described in [41].

### 2.7. State Transition Measurements Using Pulse-Amplitude-Modulated (PAM) Chlorophyll Fluorescence at Optimal Temperatures

State transitions in cyanobacterial cells were measured using a Dual-PAM-100 fluorometer (Heinz Walz GmbH, Effeltrich, Germany). Cell suspensions with equal Chl*a* concentration of 4 μg mL^−1^ were placed in a magnetically stirred, thermostated (at 30 °C) 1 cm open cuvette and dark-adapted for 15 min prior to measurements. A built-in red LED (635 nm, 8 μmol photons m^−2^ s^−1^) was used to preferentially excite PSII, while a superimposed far-red LED (720 nm, 6 μmol photons m^−2^ s^−1^) was used to preferentially excite PSI. Light intensities within the cuvette were measured using a quantum sensor with a 400–700 nm range photon response (LI-250A, LI-COR, Lincoln, NE, USA). The LI-COR registered values for the far-red light were converted to the true far-red light intensity values of the quantum sensor with a photon response in the 700–1000 nm range (MIJ-14IR, Environmental Measurement Japan, Tokyo, Japan) using a previously measured conversion relation. The illumination protocol was as follows: One minute after turning on the measuring light (620 nm, 0.22 µmol photons m^−2^ s^−1^), a 0.8 s saturating pulse (635 nm light, 3000 µmol photons m^−2^ s^−1^) was applied to determine minimal (Fo) and maximal (Fm) fluorescence levels in the dark-adapted State. After another minute, red and far-red lights were turned on simultaneously to activate photosynthesis and induce State 1. After 16 min, far-red light was turned off for 16 min to induce the State 1→State 2 transition and then turned on again for the next 16 min to induce the State 2→State 1 transition. Again, far-red light was turned off for 16 min to induce the second State 1→State 2 transition. After that, the red light was also turned off, and Fo’ was measured for another 2 min, and the measuring light was also turned off. During each 16 min illumination period, a saturating pulse was applied at the 15th minute to determine the maximal fluorescence, Fm1 (State 1) or Fm2 (State 2).

For *Arabidopsis* leaves, State transitions were measured using a MINI-PAM fluorometer (Walz, Effeltrich, Germany) equipped with two external halogen lamps (MHF-H50LR, Moritex, Saitama City, Japan) at ~23 °C. A red light (650 nm interference filter, 17 µmol photons m^−2^ s^−1^) preferentially excited PSII light, while superimposed far-red light (720 nm interference filter, 13 µmol photons m^−2^ s^−1^) targeted PSI. The illumination protocol was as follows: At 1.5 min after turning on the measuring light (650 nm, 0.03 µmol photons m^−2^ s^−1^), a 0.8 s saturating pulse (white light, 2800 µmol photons m^−2^ s^−1^) was applied, and at the 4th min, red and far-red lights were turned on. After 20 min, far-red was turned off for 20 min and then turned on again for another 20 min. At the end, both lights (red and far-red) were turned off. During each 20 min illumination period, a saturating pulse was applied at each 19th minute to determine the maximum fluorescence Fm1 (State 1) or Fm2 (State 2). The plants were dark-adapted for 1 h before measurements, which were performed on detached leaves with wet paper below.

The State transitions were characterized by their extent and kinetics. Two parameters were used: The State transition parameter qT = (Fm1 − Fm2)/Fm1 characterizes the PSII cross-section changes [11,42] and quantifies the extent of State transitions [43]. The State transition parameter qS characterizing the electron transport balance, was calculated as qS = [(Fi’ − Fi) − (Fii’ − Fii)]/(Fi’ − Fi) [44,45], where Fi and Fii are the steady state fluorescence levels in the presence of Light 1 (far-red light) in State 1 and State 2, respectively, while Fi’ and Fii’ designate fluorescence in the absence of Light 1 in State 1 and State 2, respectively. Kinetics of State 1→State 2 and State 2→State 1 transitions in *Synechocystis* and of State 1→State 2 transition in *Arabidopsis* Col-0 were best fitted to two-component exponential functions of the respective decreasing or increasing F(t) parts of State transition traces. The goodness-of-fit was judged by the statistical criteria and residual analysis provided by the OriginPro 8 (OriginLab, Northampton, MA, USA).

### 2.8. Photosynthetic Characteristics Derived from PAM Chlorophyll Fluorescence

Steady-state Chl*a* fluorescence characteristics were also estimated from the State transitions traces. The following parameters were determined: maximum quantum efficiency of PSII in dark-adapted state Fv/Fm = (Fm − Fo)/Fm; effective quantum yield of photochemical energy conversion of PSII in light State 1 and State 2, Φ_PSII_ = (Fm’ − Fs)/Fm’ [46], wherein Fm’ is Fm1 for State 1 or Fm2 for State 2, and Fs was steady state level in light State 1 (Fi) or in light State 2 (Fii’), respectively; photochemical energy quenching (qP) in State 2 was calculated using qP = (Fm2 − Fii’)/(Fm2 − Fo’) [46]. The fraction of closed (reduced) PSII reaction centers was calculated as 1 − qP.

### 2.9. Statistics

All statistical analyses were performed using OriginPro 8 (OriginLab, Northampton, MA, USA). Where appropriate, data were analyzed using a paired *t*-test with a significance threshold of *p* < 0.05. Statistical significance levels are indicated in the tables as *p* < 0.05 (*), *p* < 0.01 (**), and *p* < 0.001 (***). The *t*-test was conducted in Microsoft Excel. Data are expressed as the mean ± SD unless otherwise stated.

## 3. Results

### 3.1. Effect of the PetD-F124A Mutation on Cell Growth and Pigment Content

**Figure 4:**
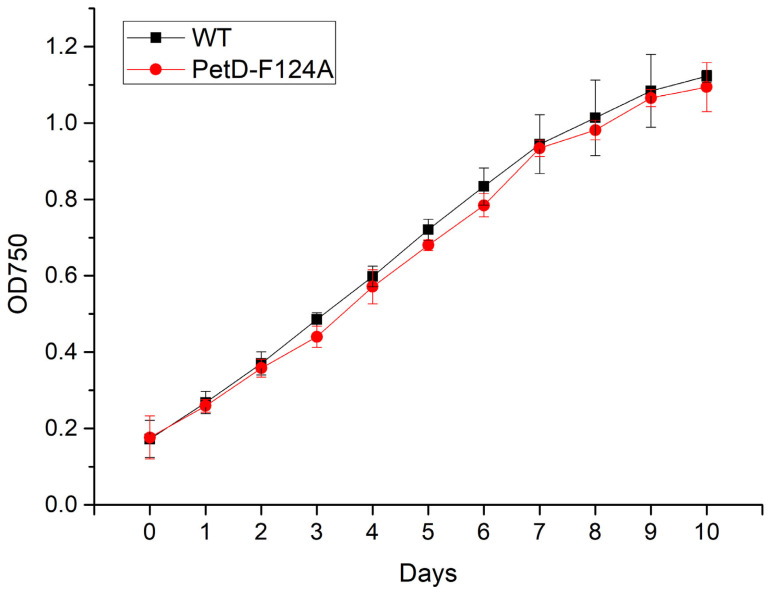
Growth curves of photoautotrophically grown *Synechocystis* WT and PetD-F124A mutant cells. Data represent the mean ± SD of three independent culture generations.

As a first step in our study, we assessed the impact of the substitution of Phe124 with Ala on the growth of *Synechocystis*. Figure 4 shows the growth curves of WT and PetD-F124A mutant *Synechocystis* cells and reveals no statistically significant difference between the WT and PetD-F124A strains, indicating that this point mutation has no marked effect on cell vitality or overall photosynthetic capacity under standard growth conditions.

**Figure 5:**
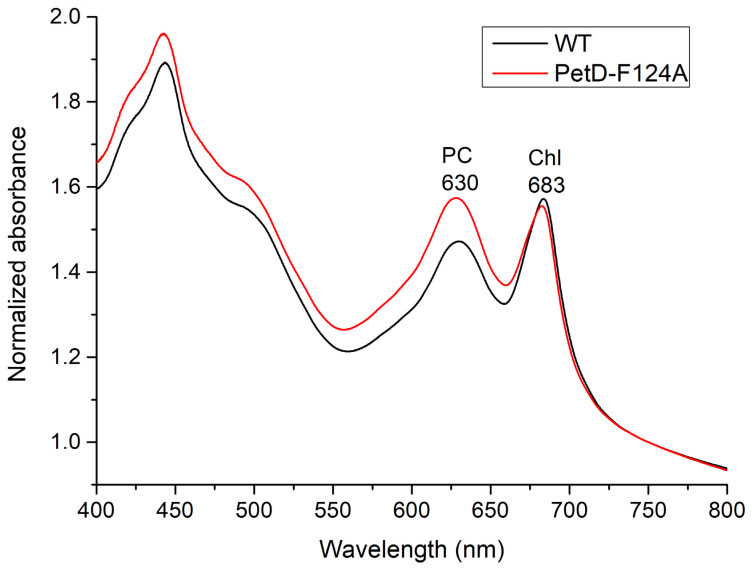
Absorption spectra of *Synechocystis* WT and PetD-F124A mutant cells, normalized to the OD at 750 nm. The spectra are the average of four independent samples.

To evaluate potential alterations in pigment content, we recorded whole-cell absorption spectra of both strains (Figure 5). The spectra show that the single Phe124Ala substitution increases the PC absorption peak (~630 nm) relative to the Chl*a* absorption peak (~683 nm).

Using the formulas in [38], we quantified molar concentrations of Chl*a* and PC, as well as their molar ratio and the *A*_630_/*A*_683_ absorbance ratio (Table 1). The mutant exhibited a 12% decrease in Chl*a* content (statistically significant, *p* < 0.001), while PC increased by ~4% (not significant). Consequently, the PC/Chl*a* molar ratio rose by 16% in the mutant, a change that was statistically significant, and driven primarily by the reduction in Chl*a*.

It has been previously established that the corrected *A*_630_/*A*_683_ absorbance ratio using the formulas in [38], can serve as an indicator of the (PBS-PSII)/PSI ratio ([47] and References therein). In agreement, the *A*_630_/*A*_683_ ratio was 19% higher in PetD-F124A compared to WT (*p* < 0.001, Table 1). Given the lack of a significant change in PC content, this increase must stem from a reduction in PSI content, while PSII content remains essentially unchanged.

These findings were collaborated by direct Chl*a* quantification via methanolic extraction [39]. After normalization to OD750, the mutant showed a 22 ± 2% decrease in Chl*a* concentration (7.07 ± 0.21 μg mL^−1^ for *PetD*-F124A vs. 9.06 ± 0.31 μg mL^−1^ for the WT, n = 3, *p* < 0.001).

**Figure 6:**
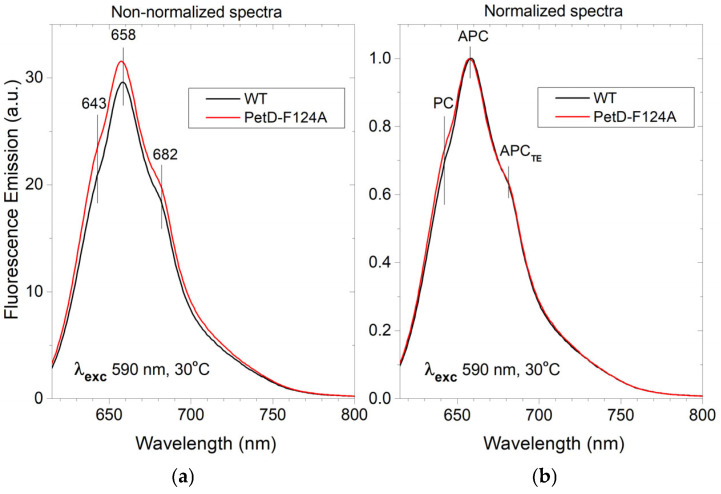
Fluorescence emission spectra of *Synechocystis* WT and PetD-F124A mutant cells upon 590 nm (PBS) excitation at 30 °C. Cells were adjusted to equal Chl*a* concentration (2 µg mL^−1^) and dark-adapted for 15 min. Representative (**a**) non-normalized and (**b**) normalized (to the maximum intensity) spectra from three independent biological repetitions are shown.

The altered pigment stoichiometry was further evident in fluorescence emission spectra recorded at 30 °C (Figure 6).

Upon PBS excitation (590 nm), spectra are dominated by allophycocyanin (APC) emission at ~658 nm, with shoulders at 643 nm (PC) and 682 nm (APC terminal emitter (APC_TE_) and PSII-bound Chl*a*) [48,49,50]. Non-normalized spectra (Figure 6a) show higher overall emission in the PetD-F124A mutant, consistent with the higher PC/Chl*a* ratio in the mutant (Table 1). Normalized spectra (Figure 6b) reveal equal relative intensities of the 658 nm (APC core) and 682 nm (APC_TE_ and PSII Chl*a*), but a statistically significant ~7% increase in the 643 nm PC shoulder (n = 3, *p* < 0.05). This suggests a modest increase in PBS rod content per Chl*a* in PetD-F124A, likely due to longer PBS rods, while the PBS core content remains unchanged.

### 3.2. PetD-F124A Mutation Does Not Alter the PSI Trimer/Monomer Ratio

Given that PSI harbors ~90% of cellular Chl*a* in Synechocystis, we investigated whether the reduced Chl*a* content in the mutant reflects changes in PSI oligomerization. CD spectroscopy is highly sensitive to pigment-protein organization, and the CD band ~510 nm specifically reports for changes in the PSI trimer/monomer ratio [51].

**Figure 7:**
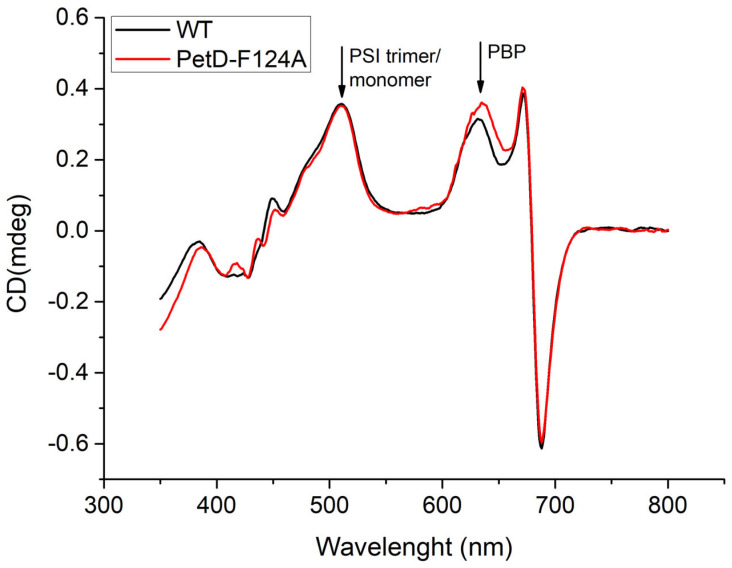
CD spectra of *Synechocystis* WT and PetD-F124A mutant cells grown at 30 °C. The main peaks (indicated by arrows) belong to PSI trimer/monomer fingerprint (~510 nm) and PBP (phycobiliproteins) band (~630 nm).

As shown in Figure 7, the CD spectra of WT and PetD-F124A strains are identical in the 510 nm region, indicating that the mutation does not affect the PSI trimer/monomer ratio. However, a 14% increase in the phycobiliprotein (PBP) band at ~630 nm [52] is observed in the mutant, consistent with the absorption data (Section 3.1). The agreement between the absorption and CD data supports the conclusion that the PBP/Chl*a* ratio is modestly increased (~14%) in the PetD-F123A mutant.

### 3.3. Thermodynamic Stability of Phycobilisomes in WT and PetD-F124A Mutant Cells

**Figure 8:**
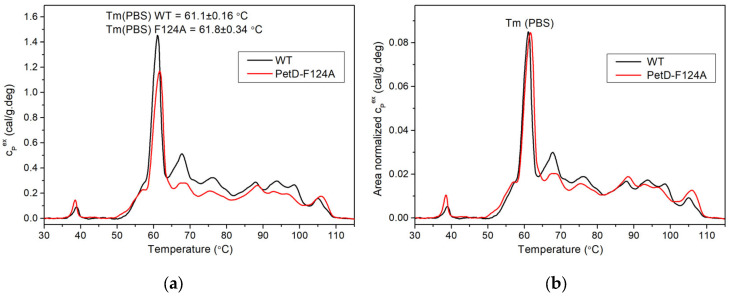
DSC thermograms of photoautotrophically grown WT (black) and PetD-F124A mutant (red) *Synechocystis* cells. (**a**) Excess heat capacity, c_P_^ex^ (amplitude of the transition) normalized to the protein content. (**b**) Same data further normalized to total scan enthalpy (area under the thermogram). Scanning rate 1 °C min^−1^. The scans are averaged from 2 to 3 independent cell culture measurements, and the main transition temperature (Tm ± SD) is indicated in (**a**).

To assess whether the altered PBS composition in the mutant affects structural integrity of the PBSs in intact cells, we employed DSC [41]. As shown in Figure 8a, the DSC thermograms of both WT and mutant intact cells are dominated by a sharp, high-amplitude thermal transition at ~61 °C, attributed to the PBSs. This transition strongly depends on the structural integrity of the PBS supercomplex and is the only transition convincingly assigned in the thermograms of whole cyanobacterial cells to date [41]. To estimate the relative contribution of the PBS transition to the total excess heat capacity of the two strains, we analyzed their area-normalized excess heat capacity scans (Figure 8b).

The thermodynamic parameters of the PBS transitions were highly similar for both strains: (I) the temperature of PBS denaturation, T_m_ (PBS) WT = 61.1 ± 0.2 °C and T_m_ (PBS) PetD-F124A = 61.8 ± 0.3 °C, corresponding to an upshift of 0.7 °C; (II) the cooperativity of the transition, expressed as the width at half height (*T*_1/2_), differed by only 0.4 °C (2.9 °C for WT and 3.3 °C for PetD-F124A); and (III) the excess heat capacity of the PBS assigned transition (Figure 8b). It should be noted that the 0.7 °C increase in T_m_ of the mutant is small relative to the standard deviation and does not reach statistical significance. Therefore, this minor shift should not be interpreted as a meaningful change in PBS thermal stability. These results indicate that the PetD-F124A mutation does not compromise the structural integrity of the PBSs in the mutant cells. The slightly higher T_m_ (PBS) of the mutant cells may be due to a larger PBS size, because shortening of the PBS rods, uncoupling of PC units, or destabilization of the PBS structure lowers T_m_ [53]. This interpretation is further supported by slightly lower cooperativity of the PBSs denaturation transition in the mutant cells, suggesting a more heterogeneous PBS size distribution. Expressed in other words, the mutant population likely contains both normal and larger PBSs, as suggested by the DSC data. The larger size of the PBSs in the mutant cells could be due to longer rods per PBS, rather than free rods, as the DSC results showed that the PBSs’ structural integrity is preserved.

### 3.4. Low-Temperature 77K Fluorescence Emission Spectra

**Figure 9:**
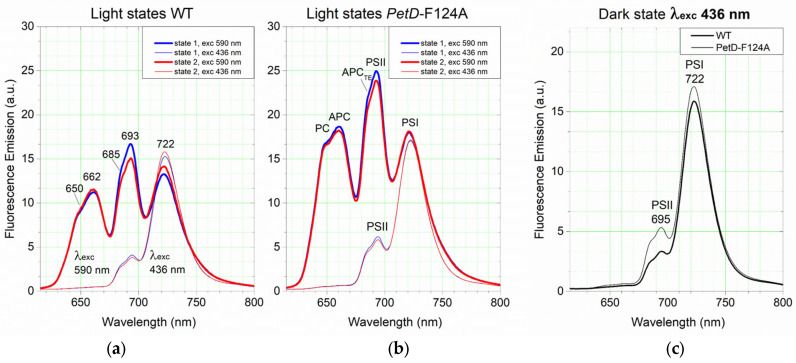
77K fluorescence emission spectra of *Synechocystis* WT and PetD-F124A mutant cells. Spectra of (**a**) Light-adapted WT cells and (**b**) PetD-F124A mutant cells in State 1 (blue lines) and State 2 (red lines) upon excitation with 590 nm (PBSs, thick lines) and 436 nm (Chl*a*, thin lines) light. (**c**) Dark-adapted WT (thick black) and mutant (thin black) cells upon 436 nm excitation. A dark state of the cells was achieved after 15 min of darkness. Light State 1 was reached after 16 min of illumination with red (635 nm, 8 μmol photons m^−2^ s^−1^) plus far-red (720 nm, 6 μmol photons m^−2^ s^−1^) light, as during Dual-PAM State transitions monitoring. Light State 2 was achieved after an additional 16 min of illumination of the cells in State 1 with red light only. The cells were adapted to the respective States after deposition on filters in disks. Still under the respective dark or light state conditions, the disks were immediately immersed in liquid nitrogen (below 0.5 s). All spectra were recorded at an equal Chl*a* content of 3 µg on the filter. The same sample was first excited at 590 nm and then at 436 nm. The spectra are not normalized or calibrated with an added external fluorophore; each spectrum is the average of measurements performed on three independent cell batches.

To quantitatively assess the impact of the PetD-F124A substitution on the PSI:PSII emission stoichiometry, energy transfer efficiency from PBS to PSs, and State transition capacity, we recorded 77K fluorescence emission spectra of WT and mutant cells. Figure 9 shows the absolute (non-normalized) fluorescence spectra of cells adapted to State 1 and State 2 upon excitation with 590 nm (preferential excitation of PBS) and 436 nm (preferential excitation of Chl*a*) (a,b) and in the dark-adapted State (c) upon 436 nm excitation. In the 590 nm (PBS) excited spectra, the shoulder at 650 nm (F650) and the peak at ~662 nm (F662) originate from PC and APC of the PBSs, respectively [54]. The shoulder at around 685 nm (F685) originates from both the APC_TE_ and PSII (CP43). The peak at ~693 nm (F693) results from the core antenna CP47 of PSII (e.g., [36,55,56]). In both the 590 nm (PBS) and 436 nm (Chl*a*) excited spectra, the peak at around 721–723 nm (F722) belongs to PSI (e.g., [57]). In the 436 nm excited spectra, the small peaks at approximately 685 nm and 695 nm are attributed to the CP43 and CP47 core antenna of PSII, respectively [36,56].

Overall, PetD-F124A exhibit higher fluorescence yield than WT at equal Chl*a* content (3 µg), consistent with its ~16% higher PC content per cell (see Section 3.1), which enhances its fluorescence.

#### 3.4.1. PetD-F124A Mutation Reduces the PSI Content

To complement the already established lower PSI content in the mutant cells using the *A*_630_/*A*_683_ absorbance ratio (Table 1, Section 3.1), we determined the PSI/PSII emission stoichiometry ratio using the F722/F695 emission ratio from 436 nm excited spectra in the dark State [47,58] (Figure 9c). This ratio was 4.76 ± 0.11 in WT and 3.22 ± 0.04 in PetD-F124A (n = 3, *p* < 0.001). Since the absorption data suggested that the PSII content remains essentially unchanged by the mutation (Section 3.1), the fluorescence-detected ~30% decrease in PSI content is similar to that determined by absorption (Section 3.1). Thus, both methods confirm the decrease in PSI content in the PetD-F124A mutant, in excellent agreement with the 22 ± 2% reduction in total Chl*a*, measured by methanolic extraction.

#### 3.4.2. Excitation Energy Transfer from PBS to PSII and PSI

To assess whether the mutation affects the efficiency of excitation energy transfer, we used several fluorescence emission ratios (Table 2). Table 2 summarizes key emission ratios upon 590 nm (PBS) excitation and the ratio of PSI emission peak at 722 nm upon 590 nm and 436 nm excitation at 77K They are used to detect differences between the two cyanobacterial strains in the efficiency of energy transfer within the PBSs (PC→APC→APC_TE_), from the PBSs to PSII, and from the PBSs to PSI.

The F662/F650 ratio indicates the energy transfer efficiency from the PC of the rods to the bulk APC of the cores of the PBSs. This ratio is statistically significantly lower in PetD-F124A than in WT by ~12% in both State 1 and State 2 (Table 2). However, the F685/F662 ratio, which indicates the energy transfer efficiency from the bulk APC to the terminal emitters APC_TE_/CP43, does not differ in the two strains, confirming intact APC-APC_TE_ connectivity. Given the facts that (I) the PC emission but not the APC emission is higher in PetD-F124A emission spectra at 30 °C (Figure 6), (II) the preserved integrity of the PBSs (Figure 8), and (III) that the energy transfer efficiency is strongly dependent on the distance (~d^−6^), the diminished efficiency of PC→bulk APC energy transfer is suggestive for longer rods in PetD-F124A than in the WT.

The F693/F685 ratio, which indicates the energy transfer efficiency from the APC_TE_ to CP47 and PSII RC, differs in PetD-F124A from that in WT only in State 1 (Table 2). This difference indicates a suppressed (by ~5%) energy coupling between PBSs and PSII in State 1 of PetD-F124A relative to State 1 of the WT, i.e., a smaller effective antenna size (~5%) of PSII in State 1 of PetD-F124A compared to WT. This can be visualized as a more destabilized/disordered PSII-PBS rows (megacomplexes) in PetD-F124A compared to the more ordered megacomplexes of WT, as shown by [59,60].

The ratio F722(λ_exc_ 590)/ F722(λ_exc_ 436) (see Table 2), can be used as a rough indicator of the relative efficiency of energy transfer from PBSs to PSI in the two states of the WT and the mutant species [61]. In WT, this ratio is slightly higher in State 2 (Table 2), consistent with the more efficient energy transfer to PSI in State 2. Notably, in PetD-F124A, this ratio is the same in both states and is much higher than in the WT. This indicates more efficient PBSs→PSI energy transfer in both States and strongly supports the view that (i) PBSs remain energetically coupled to PSI in both states and (ii) PetD is locked in a state closer to State 2 (i.e., PBSs remain bound to PSI in both states).

#### 3.4.3. Characterization of Stationary State 1 and Stationary State 2 by 77K Fluorescence Emission Spectra

**Figure 10:**
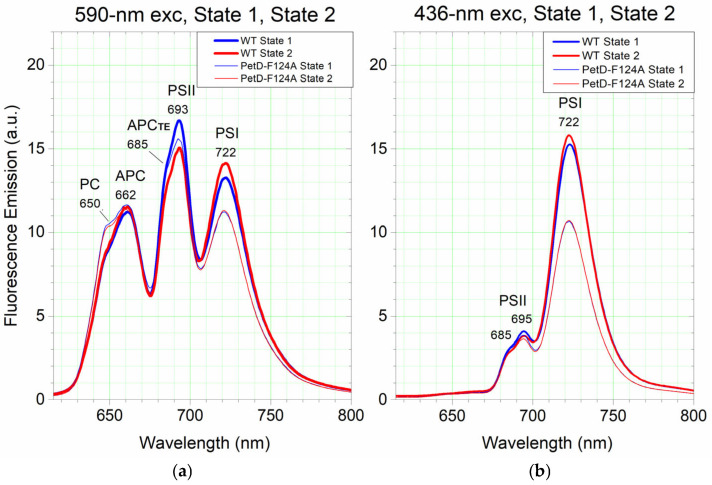
The 77K emission spectra of *Synechocystis* WT and PetD-F124A mutant cells, normalized to equal content of PSII per cell upon 590 nm (PBS) (**a**) and 436 nm (Chl*a*) (**b**) excitation. The WT spectra are the same as in Figure 9a. The PetD-F124A spectra from Figure 9b are multiplied by the ratio F695(WT)/F695(PetD-F124A), wherein F695 is the emission intensity of PSII taken from the dark State spectra upon 436 nm (Chl*a*) excitation (Figure 9c).

To compare State 1 and State 2 on a per-cell basis, we normalized the original PetD-F124A spectra, shown in Figure 9, to equal PSII content as in WT cells, using the established lower PSI content of the mutant cells through the 436 nm excited dark spectra (Section 3.4.1). All PetD-F124A spectra from Figure 9 were multiplied by the value of the F695(WT)/F695(PetD-F124A) ratio, which was estimated from the dark State spectra upon 436 nm (Chl*a*) excitation (Figure 9c). The resulting spectra (Figure 10) represent intrinsic per-cell spectra of the two strains, independent of Chl*a* concentration differences.

The 590 nm (PBS) excited spectra also provide information for the contribution of PBSs in State transitions. As seen in Figure 10a, the State 1 of WT cells is characterized by increased PSII emission (F693) with ~11.2% and concomitantly decreased PSI emission (F722) with ~6.6%, relative to the respective band intensities in State 2. The total change is 17.8%. This energy redistribution between PSII and PSI during State transitions corresponds to ~63% (11.2% from the total change of 17.8%) of the PBSs bound to PSII and ~37% (6.6% from the total change of 17.8%) of the PBSs uncoupling from PSI in State 1, and vice versa in State 2. In stark contrast, PetD-F124A cells show no changes in PSI emission intensity (F722) between State 1 and State 2 and PSII emission changes (F693) between State 1 and State 2 are only half of those in WT (5.7% vs. 11.2%). Thus, the total extent of State transitions is reduced threefold (5.7% vs. 17.8%). This confirms that the PetD-F124A mutation severely impairs State transitions, primarily by abolishing PBS-PSI uncoupling and partially suppressing PBS-PSII coupling.

The 436 nm (Chl*a*) excited spectra provide information for the contribution of membrane reorganizations in State transitions. As seen in Figure 10b, the same trend of differences between the WT and PetD-F124A spectra is observed, similar to that at 590 nm (PBS) excitation (Figure 10a). However, the extent of the registered differences between the State 1 and State 2 spectra is much smaller than that between the PBS excited spectra. For this reason, we propose that the observed differences upon Chl*a* excitation may result from PBS-redistribution-induced membrane reorganizations in State 1 and State 2.

In Figure 10, it is also seen that in State 2, upon PBS excitation, the emission ratio F722(WT)/F722(PetD-F124A) is 1.262 (a), but upon Chl*a* excitation (b) it is higher (1.481) with 17%. In State 1 spectra, upon both excitations, the PSI emission of PetD-F124A is equal to that in State 2, whereas that of WT decreases by 6.7% in State 2. The fact that there is no decrease in PSI fluorescence in State 2 of PetD-F124A relative to that in State 1 indicates that there is no energy decoupling of PBSs from PSI in the mutant. In other words, the PetD-F124A mutant has consistently bound PBS in both light States, and the PBS-PSI coupling in PetD-F124A is stronger than that in WT cells; the PSI of PetD-F124A remains bound to PBS in State 2, even when the PSI is preferentially excited with far-red light (light 1). This view was supported by the observation that low-temperature fluorescence spectra of PetD-F124A do not show a concomitant increase in PSI emission when PSII emission decreases in State 2 (Figure 9).

### 3.5. Effects of PetD-F124A Mutation on Photosynthetic Parameters and State Transitions (PAM Fluorometry)

**Figure 11:**
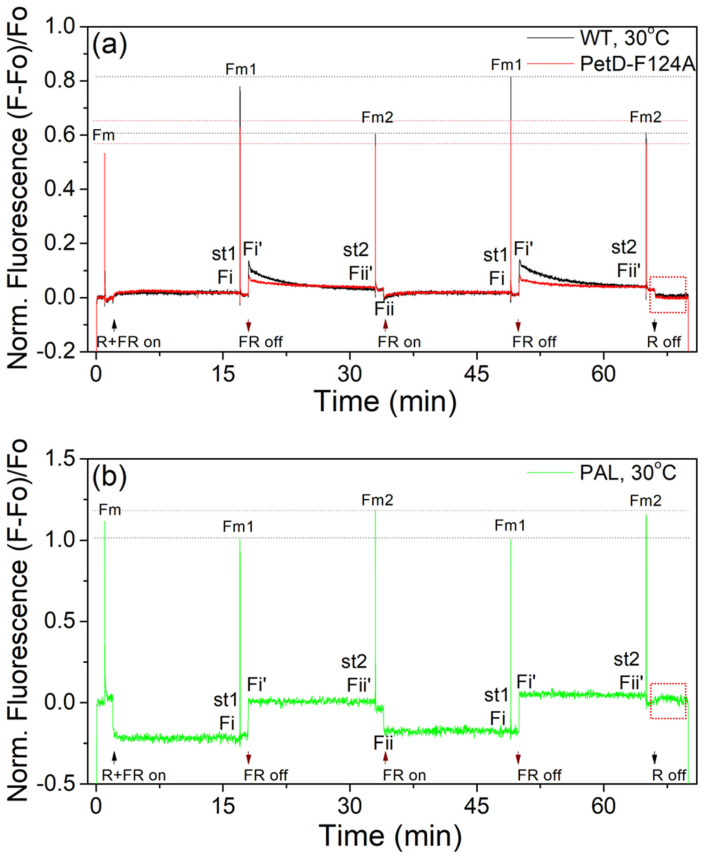
Light-induced State transitions in WT, PetD-F124A mutant (**a**) and PAL (phycobilisome-less) mutant (**b**) cells of *Synechocystis* at 30 °C measured with Dual-PAM 100. The cells were dark-adapted for 15 min. State 1 was induced by simultaneous illumination with red (R, 635 nm, 8 μmol photons m^−2^ s^−1^ preferentially exciting PSII) and far-red (FR, 720 nm, 6 μmol photons m^−2^ s^−1^ preferentially exciting PSI) lights. Transition to State 2 was induced upon switching off the FR light. Fm, Fm1, and Fm2 indicate the maximal fluorescence in dark-, State 1- and State 2-adapted states, respectively, in response to a saturating pulse (0.8 s, 3000 μmol photons m^−2^ s^−1^). Fi and Fii denote the fluorescence in the presence of PSI light (FR) in State 1 and State 2, respectively, while Fi’ and Fii’ denote the fluorescence in the absence of PSI light in State 1 and State 2, respectively. The traces are normalized to Fo = 0 and represent the mean of three independent measurements on 15-day-old PAL mutant cells with a Chl*a* concentration of 2.5–3 µg mL^−1^. The dotted red boxes indicate the absence (**a**) and presence (**b**) of post-illumination fluorescence rise.

To characterize the impact of the PetD-F124A mutation on the extent and kinetics of State transitions, we performed real-time measurements at 30 °C using a Dual-PAM-100 fluorometer (Figure 11a). PAL mutant (Figure 11b) was included as a negative control for the contribution of PBS dynamics during State transitions (Figure 11a). Table 3 summarizes the measured photosynthetic and State transitions parameters.

#### 3.5.1. The PetD-F124A Mutation Does Not Inhibit Electron Transport Processes

The parallel increase in both Fo and Fm in the mutant (Table 3)—without a change in Fv/Fm—indicates that Fo is higher in PetD-F124A because of the larger PBS/Chl*a* ratio. A contribution from uncoupled PBSs can be excluded because Fm, where the fluorescence of PBSs does not contribute, is also enhanced. Table 3 also shows that both species have equal Fv/Fm, indicating that the mutation does not inhibit linear electron transport. The slightly reduced Φ_PSII_ of the PetD-F124A mutant in both States relative to WT (Table 3) reflects the smaller PSII antenna cross-section in PetD-F124A. (Section 3.4.3). In State 2, the PQ pool redox state of the mutant (1-qP) is slightly more reduced than in WT, but in both states, the PQ pool is oxidized, because the light intensity is low. Notably, neither WT nor PetD-F124A exhibits a post-illumination fluorescence rise (Figure 11a, red box), unlike the PAL mutant (Figure 11b). This transient rise reflects non-photochemical PQ reduction via NDH-mediated cyclic electron flow around PSI (CEF-PSI) [62,63]. Therefore, this result shows that the mutation has no detectable effect on CEF under these conditions.

#### 3.5.2. The PetD-F124A Mutation Selectively Suppresses State Transitions

The most striking difference between WT and mutant is the markedly reduced Fm1 and Fi’ in PetD-F124A (Figure 11a), directly reflected in ~50% lower qT and qS values (Table 3). The twofold reduction in qT (Table 3) arises primarily from a greater decrease in Fm1 (State 1) in the mutant, while Fm2 (State 2) remains similar. This indicates that State 1 is severely impaired, while State 2 is largely preserved—consistent with permanent PBS-PSI coupling in both states (Section 3.4.3). The 44% lower qS value shows that the mutant recovers only half as effectively as WT from light-quality changes.

#### 3.5.3. The Mutation Selectively Delays the Induction Phase of State 2→State 1 Transitions

The t_ind1→2_ and t_ind2→1_ parameters in Table 3 present the induction times of the two transitions, estimated from Figure 11a. The induction time for State 1→State 2 transition, t_ind1→2_, is the time needed to reach the Fi’ maximum from the stationary Fi level (i.e., when the PQ pool changes its redox state from oxidized to over-reduced), while the induction time for State 2→State 1 transition, t_ind2→1_, is the time needed to reach the Fii minimum from the stationary Fii’ level (i.e., when the PQ pool changes its redox state from oxidized to over-oxidized) ([8,9,64], Figure 11a). These induction times provide information on how fast the signal transduction system transmits the light-quality-induced changes in the PQ pool redox state. As is seen in Table 3, while t_ind1→2_ is unchanged (3.9 vs. 4.2 s), t_ind2→1_ is tripled in the mutant (1.3 vs. 4.2 s, *p* < 0.05). We interpret this as a loss of synchronization between the PQ pool redox changes and the conformational signaling at the cyt*b*_6_*f* transducer. The absence of an aromatic ring in the mutant at position 124 (see Figure 1) likely slows the signal transmission for the change in the redox state of the PQ pool from oxidized to over-oxidized state to the primary effectors—thylakoid lipids—that trigger PBS detachment from PSI [8].

#### 3.5.4. The PetD-F124A Mutation Selectively Alters the Kinetics of State Transitions

State transition kinetics were analyzed by fitting fluorescence transients to exponential models (Table 4). The PAL mutant (Figure 11b), which lacks PBSs, was used as a negative control for the effect of PBSs on the State transitions kinetics. In the absence of PBSs (Figure 11b), we observe an immediate rise and drop in fluorescence, with no restoration of PQ pool redox poise. Only when PBSs are present in the WT (Figure 11a) do we observe the kinetics of State transitions and the restoration of PQ pool redox poise. Comparison with PAL strongly supports the notion that the observed changes in the State transitions traces in the WT and PetD-F124A (Figure 11a) are due to the presence of the PBSs, confirming that the PBS movement is a prerequisite for observing light-induced state transitions [65,66], and explains the absence of light-induced state transitions in the PAL mutant [67,68]. Furthermore, as seen in Table 4, the kinetics of fluorescence decrease during the State 1→State 2 transition and increase during State 2→State 1 transition in WT and PetD-F124A (Figure 11a) are best fitted by two-exponential components, thus demonstrating that the PBSs are not the sole protein complex that moves during State transitions.

As seen in Table 4, the fluorescence decrease during the State 1→State 2 transition is characterized by fast (t_1/2_1, seconds) and slow (t_1/2_3, two orders of magnitude longer than t_1/2_1) halftimes in both WT and the PetD-F124A mutant. The relative amplitudes of these components clearly show that the State 1→State 2 transition is dominated by the slow component (A2 ~25% vs. A3 ~75%, Table 4). The F124A substitution does not change the halftimes t_1/2_1 and t_1/2_3. It changes only the amplitude A3 of the slow time-component to only 40% from that of the WT (A3 of mutant ~30% vs. A3 of WT~74%). This reduces the amplitude-averaged t_1/2_*av* of the State 1→State 2 transition in the PetD-F124A relative to WT (Table 4). These results indicate that while the fast reorganization in WT and PetD-F124A is similar, only 40% of the slow reorganization in the WT takes place in the mutant during the State 1→State 2 transition. Based on comparisons with the PAL mutant and the identified MGDG-resorting around cyt*b*_6_*f* during the induction of this transition [8], the fast component with amplitude A1 can be assigned to the MGDG-PBS detachment and movement as proposed by the HMM model [9]. The much smaller A3 of the mutant (only 40% of that of the WT) of the slow time-component indicates that the mutant in its State 1 is much closer to State 2, and a much smaller extent of reorganization is required to reach the State 2 organization. Note that in the cyanobacterial WT, the amplitude A3 of the slow time component t_1/2_3 of the State 1→State 2 transition is much higher (~70%) than the A1 (~30%). As will be seen below (Section 3.5.5), the A3 can be assigned to reorganizations involving the membrane pigment-protein complexes such as PSII and PSI, because the same time scales are the reorganizations in the cyanobacterial and plant State 1→State 2 transition. Therefore, this result indicates that in the presence of PBSs, membrane-related reorganization processes (A3 and t_1/2_3) are a significant contributor to cyanobacterial State transitions.

The fluorescence increase during the State 2→State 1 transition is characterized by fast (t_1/2_1, seconds) and middle (t_1/2_2, one order of magnitude longer than t_1/2_1) halftimes in both WT and the PetD-F124A mutant (Table 4). However, in contrast to the State 1→State 2 transition, during the State 2→State 1 transition, the F124A substitution enlarged both halftimes, but does not change their amplitudes. Thus, just as the induction phase of the State 2→State 1 transition is strongly delayed by the absence of the aromatic ring residue at position 124 in PetD (Table 3, Section 3.5.3), both fast and middle time reorganizations are also strongly delayed. Therefore, the amplitude-averaged t_1/2_*av* of the State 2→State 1 transition in the PetD-F124A is larger relative to that of the WT (Table 4). Note that the two transitions have different characteristics, consistent with the different lipid-resorting that induce them. The fast component during the State 2→State 1 transition can be ascribed to the DGDG depletion, which destabilizes the PBS-PSI interactions [8]. The middle component can be ascribed to the DGDG-depletion-induced mutual membrane protein reorganization post-detachment, because A1 = A2 and membrane reorganizations in this time range are observed in plants (Section 3.5.5, below).

These findings confirm that Phe124 aromatic ring—whose rotation correlates with the cyt*b*_6_*f* hydrophobic thickness [8,9]—accelerates State 1 induction by promoting DGDG sorting around cyt*b*_6_*f* and PBS release from PSI (see Figure 1).

#### 3.5.5. Comparison of the Cyanobacterial and Plant State Transitions Kinetics

**Figure 12:**
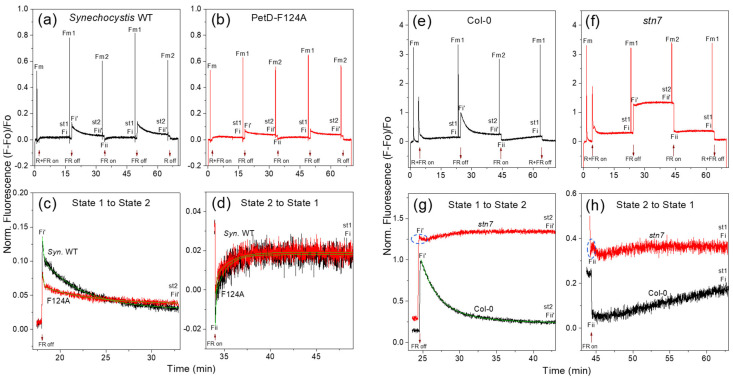
Comparison of fluorescence changes during State transitions in cyanobacterial *Synechocystis* WT (**a**,**c**) and PetD-F124A mutant (**b**,**d**) cells with those in plant *Arabidopsis* WT (Col-0) (**e**,**g**) and its *stn7*-mutant (**f**,**h**). The green curves in panels (**c**,**d**,**g**) represent two-exponential component fitted curves of the cyanobacterial State 1→State 2 (**c**) and State 2→State 1 (**d**) and plant Col-0 State 1→State 2 (**g**) transitions. The dashed blue ovals in panels (**g**,**h**) highlight the initial fluorescence changes in the plant *stn7* mutant that closely resemble those in the respective cyanobacterial State transitions (**c**,**d**)—suggesting a kinase-independent component of State transitions.

To test the predictions of the HMM model for at least two-component State transitions kinetics in both cyanobacterial and chloroplast State transitions, we analyzed this kinetics in the WT *Arabidopsis* (Col-0) plant and its LHCII kinase mutant *stn*7. We compared them with those in *Synechocystis* (Figure 12 and Table 5).

The State 1→State 2 transition in Col-0 WT also follows two-exponential fluorescence decrease kinetics (Table 5). However, unlike in cyanobacteria (Table 4), it lacks a fast component (t_1/2_1 ~8 s) and instead exhibits a middle (t_1/2_2 ~70 s) and slow (t_1/2_3 195 s) time components, the latter comparable to the cyanobacterial slow (t_1/2_3 ~210 s) component. Notably, the ~70 s middle component aligns with the time required for full phosphorylation of the LHCII in the alga *Chlamydomonas* (<2 min) [26]. In *Arabidopsis*, the middle component dominates the transition (amplitude A2 ~70%), with the slow contributing ~30% (A3) (Table 5). By contrast, in cyanobacteria, the slow component dominates the transition (A3 ~70%), reflecting the MGDG-PBS detachment-induced membrane-protein reorganization. The fast component (A1 ~30%) reflects the MGDG-PBS-PSII destabilization and MGDG-PBS coordinated movement (Table 4, Section 3.5.4).

However, the *stn7* mutant exhibits an initial rapid drop in fluorescence at the onset of State 1→State 2 transition (the dashed blue oval in Figure 12g), followed by partial recovery and stabilization. This transient drop—also observed in other *stn7* studies [69,70] but previously uninterpreted—provides direct evidence of a kinase-independent State transition component. We attribute this to the same MGDG-detachment-induced destabilization of the more ordered PSII dimer arrays in grana stacks—mechanistically analogous to MGDG-PBS detachment in cyanobacteria. However, due to the dense packing of PSII-LHCII supercomplexes in plant grana, MGDG diffusion is significantly slower than in the more fluid cyanobacterial thylakoids. In WT plants, the STN7 kinase accelerates fluorescence decrease, indicating that MGDG-LHCII detachment from the PSII-LHCII supercomplexes occurs concomitantly with LHCII phosphorylation by the STN7 kinase. This supports the HMM model proposal [8] that during State 2 induction (I) MGDG sorts around cyt*b*_6_*f*; and (II) STN7 kinase is recruited to and segregated around cyt*b*_6_*f*. Notably, the relative amplitudes of the two components (A2 ~70% and A3 ~30%, Table 5) are very close to the recently estimated 60% contribution of the STN7-dependent LHCb2 phosphorylation and the 40% contribution of STN7-dependent but LHCb2 phosphorylation-independent unknown component [71].

The initial phase of the State 2→State 1 transition in the *stn7* mutant also resembles the cyanobacterial State 2→State 1 transition with its fast initial increase—highlighted by a dashed blue oval in Figure 12h. This supports the HMM proposal [8] that depletion of DGDG from the bulk lipid phase destabilizes PSI-antenna supercomplexes, leading to reduced electron withdrawal from the transport chain and a consequent fast rise in fluorescence. In WT *Arabidopsis*, fluorescence increases almost linearly (Figure 12h), reflecting constitutive phosphatase activity [13]. The phosphatase dephosphorylates p-LHCII, but its diffusion to PSII is slower than the mobility of p-LHCII itself [72]. Unlike in cyanobacteria, which exhibit clear two-exponential kinetics, the plant State 2→State 1 transition appears monophasic, dominated by dephosphorylation dynamics. Thus, while the State 1→State 2 transition in plants involves combined lipid-driven and kinase-dependent middle and kinase-dependent-membrane-protein reorganization slow components, the State 2→State 1 transition is primarily governed by phosphatase activity. Nevertheless, the initial kinase-independent signal in *stn7* confirms a conserved biophysical core across oxygenic photosynthetic organisms. Further detailed investigation of the plant State 2→State 1 kinetics is warranted.

## 4. Discussion

### 4.1. Cytochrome b_6_f Complex Is Involved in Cyanobacterial State Transitions

Our in vivo findings confirm a novel regulatory site, Phe124*^fg^*^-loop-PetD^, as a critical element in the signaling pathway for State transitions in cyanobacteria [9]. Its involvement in signaling and regulation of State transitions directly confirms that cyt*b*_6_*f* is part of the signaling pathway for cyanobacterial State transitions. Through comparative studies of the specifically generated cyanobacterial point mutant (PetD-F124A) and its WT, *Synechocystis*, using the Dual-PAM-100 apparatus in real-time, and with low-temperature (77K) fluorescence at stationary conditions, a quantitative characterization of the effect of the targeted mutation in the cyt*b*_6_*f* on the properties of State transitions was accomplished. It was established that the point mutation strongly suppresses the ability to undergo State transitions (qT, Table 3) and to rebalance the electron transport rate after the changes in the spectral quality of light (qS, Table 3). The 77K fluorescence has shown that this is expressed as diminished PSII-PBS coupling in State 1 and unchanged PSI-PBS coupling in States 1 and 2 (Figure 10, Section 3.4.3). The mutation selectively and strongly suppresses the transition to State 1 (Figure 11a and Figure 12a–d) and delays the induction phase and the progression of the transition to State 1 (Table 3 and Table 4). Thus, the mutation significantly impairs the State 2→State 1 transition, while State 2 establishment remains largely unaffected. These data provide direct functional evidence that cyt*b*_6_*f* is an active participant in the signaling network governing cyanobacterial state transitions, overturning prior inhibitor-based conclusions [15].

**Figure 13:**
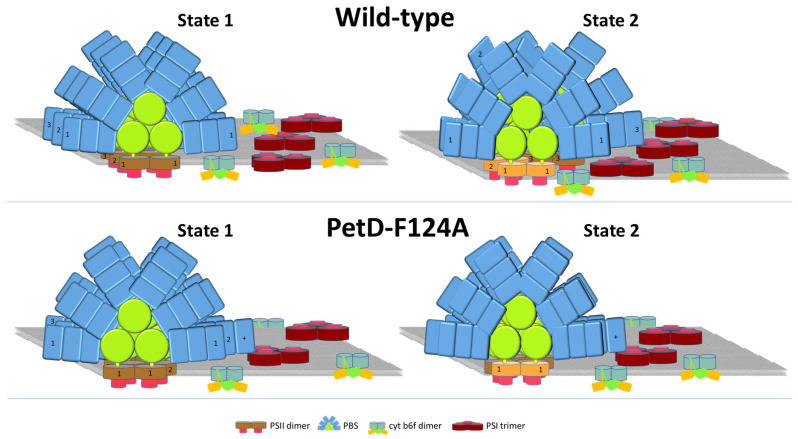
Schematic representation of the proposed membrane organization of *Synechocystis* WT and PetD-F124A mutant in States 1 and 2, based on the obtained results in the present research. State 1 of PetD-F124A is more disordered than WT, because the energy transfer from APC_TE_ to CP47/PSII-RC is diminished, and some PBS rods are longer and are bound to PSI. State 2 of PetD-F124A is similar to that of WT.

Based on the obtained results in the present research, Figure 13 provides a schematic representation of the proposed membrane organization of *Synechocystis* WT and PetD-F124A mutant in States 1 and 2, consistent with the finding that in State 2, the PBS rods, but not the PBS-core, can bind to the PSI trimer in State 2 [30].

The results obtained in the present work are important because, first, they provide direct functional evidence, based on site-directed mutagenesis data, interpreted with atomic-resolution models, that the cyt*b*_6_*f* participates in the cyanobacterial State transitions. In this context, our results confirm the recently reported *ΔPetN* mutation study and reach the same conclusion [22]. Second, our results validate in vivo that the Phe124*^fg^*^-loop-PetD^ residue is crucial for cyanobacterial State transitions, and its conformational dynamics strongly facilitate the State2→State1 transition. It is thus demonstrated that the same amino acid residue in cyt*b*_6_*f* participates in both cyanobacterial (this work) and chloroplast State transitions [25]. Third, these findings support the proposals of the HMM model [8,9] as detailed below.

### 4.2. The Hydrophobic Mismatch Model for Cytb_6_f-Driven State Transitions Is Supported In Vivo

The HMM model is based on analyses of the X-ray and cryo-EM structures of cyt*b*_6_*f* and cyt*bc*_1_, combined with lipidomic data [8,9]. The present study provides in vivo confirmation of the model’s key predictions:

1. *Role of Phe/Tyr124^fg-loop-PetD^ in modulating cytb_6_f hydrophobic thickness*. The main part of the HMM model is that the Phe/Tyr124*^fg^*^-loop-PetD^ modulates the hydrophobic thickness of cyt*b*_6_*f*. The fact that the PetD-F124A substitution selectively and strongly delays the induction phase and the progression (kinetics) of the State 2→State 1 transition (Figure 11a, Table 3, Section 3.5.3 and Section 3.5.4) confirms the role of this residue in reaching cyt*b*_6_*f* conformation with minimal hydrophobic thickness for transition to State 1. The data also suggest that the substitution Phe124Ala suppresses the mutant’s ability to undergo normal transition to State 1, as in the WT. The mutant is defective in PBS’s ability to dissociate from PSI during the transition to State 1, leading to reduced PBS binding to PSII in State 1 conditions. This defect is due to the absence of an aromatic ring at the substituted residue, which precludes the ability of the cyt*b*_6_*f* to reach a conformation with minimal hydrophobic thickness during the induction phase of transition to State 1.

2. *Functional separation of electron transfer and signal transduction.* Vladkova (2016) [9] suggested that the conformational dynamics of Phe/Tyr124 during State transitions do not interfere with the main function of cyt*b*_6_*f*—proton-coupled electron transfer from PSII to PSI, and in a subsequent study [8] demonstrated that these two functions are separated in different time domains (seconds and milliseconds, respectively). The present results (Table 3) confirm these predictions by demonstrating that the electron transfer and regulation/signal transduction functions of the cyt*b*_6_*f* are separated into different domains of the protein. We show that the PetD-F124A point mutation does not directly affect linear or cyclic electron transport (Table 3, Figure 11) but can uncouple and hamper the signal transduction (Table 4). Even though the binding of PQH_2_ at the Qp-site of cyt*b*_6_*f* is not inhibited by the mutation, the signal transduction is striated, evidenced by the suppressed extent and selectively modified induction and kinetics of State transitions (Table 2 and Table 3, Figure 11).

3. *Lipids as primary effectors of signal transmission to antenna-photosystems supercomplexes.* The HMM model is also confirmed that the lipids are the primary effectors for the signal from the cyt*b*_6_*f* to the antenna-photosystems supercomplexes for their destabilization and reorganization [8]. The similar drop in fluorescence at the onset of State 1→State 2 transition and the similar rise in fluorescence at the onset of State 2→State 1 transitions in the *stn7* plant mutant, as in the cyanobacterium (Figure 12), supports the suggestion of the HMM model [8] that the signal transduction from the PQ pool to the antenna and the photosystems is transmitted by the lipids. In the WT plant, this lipid activity is combined with the segregation of kinase around cyt*b*_6_*f*, its activation after its binding to cyt*b*_6_*f*, and the phosphorylation and movement of MGDG-P-LHCII, characterized by the first time component of fluorescence decrease during State 1→State 2 transition (Table 5).

4. *Two-exponential kinetics confirm a multi-step transition mechanism.* The two exponential kinetics of cyanobacterial State transitions (Table 4) and the plant State 1→State 2 transition (Table 5) reported in the present work support the suggested at least two-component State transitions kinetics in [8]. In the cyanobacterial State transitions, we observe the proposed MGDG-PBS detachment and movement, ascribed to the fast component (t_1/2_1 ~8-3 s). In the plant, we see the concomitant proceeding of the proposed MGDG-LHCII restructuring with the activity of the STN7 kinase. The first component is ascribed to the MGDG-LHCII detachment with the concomitant phosphorylation and movement of the MGDG-p-LHCII complexes (t_1/2_2 ~80 s, Table 5). Chuartzman et al. (2008) [73] first suggested that the lipid–antenna complexes can move together. Although the State 1→State 2 transition in plants has been suggested to be a two-component process [69,74], the present work is the first that report two exponential fits of this transition. As noted in Section 3.5.5, the established components resemble those already reported in LHCII kinases investigations [26,71]. Based on the amplitudes of the two time-components in the *Synechocystis* WT, during the State 1→State 2 transition, the movement of MGDG-PBS is the first contributor to the kinetics of State transitions in cyanobacteria (A1 ~30%, Table 4), followed by the membrane reorganizations of the MGDG-PBS-PSs complexes, which are also PBS-dependent, and dominate the State transition kinetics (A3 ~70%, Table 4). In *Arabidopsis*, the movement of MGDG-P-LHCII is the main contributor, as the A2 amplitude represents ~70% (Table 5), while that for mutual readjustment of the MGDG-P-LHCII-PSI-LHCI is ~30% (Table 5). As noted above (Section 3.5.5), the plant State 2→State 1 transition kinetics require future clarification.

5. *Essential role of PBSs and MGDG in fluorescence dynamics and restoration of PQ pool redox state.* The fact that we did not observe fluorescence decrease during the State 1→State 2 transition and fluorescence increase during the State 2→State 1 transition in the cyanobacterial PAL mutant (lacking PBSs) (Figure 11b) confirms the HMM model suggestion that the MGDG-PBS complexes move together, because otherwise we should observe fluorescence changes due to the destabilization of the PSII dimer rods. Our experiments with the PAL mutant also support the view that the MGDG lipids are not bulk, but those that stabilize PBS-PSs interaction. As suggested in [8], destabilization of the binding of the non-bilayer-forming lipid MGDG will influence the binding of the peripheral (PBS) and integral (LHCII) membrane proteins by altering the lateral pressure profile [75]. The detachment of MGDGs from the ordered PBS–PSII megacomplexes in State 1 (Figure 13) will destabilize these megacomplexes, decreasing the energetic connectivity between the antenna complexes of PSII-RC that was stabilized by the lateral pressure exerted by the surrounding MGDG lipids [76,77].

Finally, the findings based on the comparison with the PAL mutant (Figure 11) not only perfectly show how the PBSs participate in State transitions, but also that their physiological role is to allow the light-induced State transitions to occur, leading to the restoration of the PQ pool redox state to that before the light quality changes. This is the first clear demonstration regarding the previously discussed unclear physiological role of the PBSs (e.g., in [4]).

Future validation suggestion for the HMM model would be (1) the X-ray crystallography or cryo-EM of the *Synechocystis* WT and PetD-F124A mutant. This would show exactly how the substitution affects the protein’s folded structure and will help to illustrate the observed functional changes at an atomic level; (2) Time-resolved (in the time region of seconds) cryo-ET of *Synechocystis* during the induction phases of State 1 and State 2 transitions to register the conformational dynamics of cyt*b*_6_*f*; MD simulations and lipid mutants are other approaches that could support the HMM-model are suggested in [8].

### 4.3. The HMM Model Explains Why the Nature of the Phe/Tyr124 Substitution Determines the Preferred State in Mutant

The Phe*^fg^*^-loop-PetD^ residue is evolutionarily conserved in cyanobacteria, plants, and some algae, whereas Tyr*^fg^*^-loop-PetD^ is found in ten algal species [9]. Both residues are functionally equivalent in State transitions [9,25]. Critically, it is the aromatic ring—with its ability to adopt distinct orientations relative to the membrane-water interface (Figure 1)—that correlates with the hydrophobic thickness of the cyt*b*_6_*f* and enables interaction with the corresponding residue in the kinase [9]. Consistent with this, the Tyr124Phe substitution in algae does not impair state transitions [25]. An intriguing question arises: why does substituting Tyr124*^fg^*^-loop-PetD^ with Lys in the alga *Chlamydomonas* lock the mutant in a state closer to State 1 [25], whereas in the present work, in *Synechocystis*, substituting Phe124*^fg^*^-loop-PetD^ with Ala traps the mutant closer to State 2? In the former, the antenna (LHCII) cannot detach from PSII under State 2-inducing light; in the latter, the antenna (PBSs) cannot detach from PSI under State 1-inducing light. The HMM model [8,9] provides a coherent explanation for these observations.

Lys is a hydrophilic, large, basic residue, expected to orient toward the aqueous, n-side of the membrane, but not inside the lipid bilayer [78]. This conformation resembles that of Phe124 in the cyt*b*_6_*f* when the PQ pool is fully oxidized, and the cyt*b*_6_*f* adopts a conformation with minimal hydrophobic thickness (Figure 1b). However, unlike Phe, Lys cannot rotate or insert into the membrane to stabilize the conformation associated with a more reduced PQ pool, and increased hydrophobic thickness of cyt*b*_6_*f*; therefore, MGDG is not needed to sort around cyt*b*_6_*f*. This precludes the activation of the Stt7 kinase, because the cyt*b*_6_*f* cannot possess the necessary conformation for MGDG sorting around cyt*b*_6_*f*, which is required to segregate the kinase around cyt*b*_6_*f*, to bind it, and to activate it, concomitantly with the destabilization of the PSII-LHCII megacomplexes by the detachment of MGDG-LHCII from them. Therefore, the mutant remains locked in State 1.

In contrast, Ala is a nonpolar, minimal, aliphatic amino acid residue that preferentially partitions into the membrane core, mimicking the buried conformation of Phe124 at optimal (Figure 1a), and a more reduced PQ pool redox state [8]. Even to rotate, Ala is too short and cannot reach the Ile211^cyt*b*6^, which corresponds to the fully oxidized PQ pool (Figure 1b). Therefore, cyt*b*_6_*f* cannot adopt this conformation, characterized by its minimal hydrophobic thickness d_P_. This leads to DGDG’s inability to sort and bind to cyt*b*_6_*f.* The automatic DGDG depletion of the bulk lipid phase, which is necessary to destabilize the PSI [79] and its interactions with the PBS rods, is precluded. Therefore, the mutant remains with PBS bound to PSI in both State 1- and State 2-inducing conditions, closer to the State 2 organization of the WT.

### 4.4. The Inability of the Synechocystis PetD-F124A Mutant to Detach PBSs from PSI During State 2→State 1 Transition Triggers Compensatory Adjustments

Several lines of evidence in the present work (Table 1 and Table 2, Figure 5, Figure 6b, Figure 7 and Figure 8b, Section 3.4.1) indicate that, compared to the WT, the PetD-F124A mutant exhibits longer PBS rods and a ~20–30% reduction in the PSI/PSII stoichiometry ratio, primarily due to decreased PSI content. For the first time, we demonstrate that a single point mutation in cyt*b*_6_*f* can profoundly affect the biogenesis of cyanobacterial pigment-protein complexes. The observed compensatory changes are induced by the inability of the PetD-F124A mutant to detach the PBSs from PSI under State 1-inducing conditions, rather than by PQ pool overoxidation. The PQ pool in the mutant and the WT is practically equally oxidized (Table 3). Because PBS-PSI interactions cannot be disrupted in the mutant, and therefore the mutant PSI is expected to have an enhanced photosynthetic rate in both states, the compensatory response to the mutation is the reduced number of PSI and elongated PBS rods, because at lower PSI content (and therefore the longer distance between the PSI and the PBSs), the PBS rods remain bound to PSI.

### 4.5. Phe124^fg-loop-PetD^ as a Master Regulatory Site for Short- and Long-Term Photosynthetic Regulation

Although our absorption, fluorescence, CD and DSC data are indirect with respect to the PSII:PSI stoichiometry and the PBS rod size, the registered changes are statistically significant and reliable. Taken together with the state transitions data, they position Phe124*^fg^*^-loop-PetD^ as a master regulatory site coordinating both short- (State transitions) and long-term (PSII:PSI stoichiometry, PBS size) adaptive responses of cyanobacteria to the spectral quality of low-intensity light. While prior studies implicated other cyt*b*_6_*f* subunits: PetA [22], PetB [80], or the Qp-site [67] in long-term regulation, we now identified Phe124*^fg^*^-loop-PetD^ as a critical residue governing PSI abundance and PBS rod size (Section 3.1, Section 3.2, Section 3.3 and Section 3.4.1). This suggests a unified signaling pathway in which cyt*b*_6_*f* conformation [8,9])—modulated by Phe124 dynamics—controls both immediate lipid-mediated antenna redistribution and downstream gene expression, possibly via redox-sensitive kinases such as Hik2 in cyanobacteria or its chloroplast homolog CSK [81]. Future work should test whether these kinases physically or functionally interact with the PetD *fg*-loop.

## 5. Conclusions

This study represents the first in vivo validation in cyanobacteria of the function of a single amino acid residue, Phe124*^fg^*^-loop-PetD^, predicted from analyses of X-ray crystal structures. It also provides, for the first time, direct functional evidence, based on mutagenesis data and interpreted using atomic-resolution models, that the cyt*b*_6_*f* is involved in cyanobacterial State transitions. Our data support the key predictions of the HMM model for cyt*b*_6_*f*-driven State transitions: (1) Phe124 plays a critical role in modulating the transition to State 1, through the conformational flexibility of its aromatic ring and its impact on the hydrophobic thickness of cyt*b*_6_*f*; (2) the separation of electron transfer and the regulatory/signal transduction functions of the cyt*b*_6_*f* into different domains of the protein; (3) the lipids serve as primary effectors transmitting the signal from cyt*b*_6_*f* to the antenna-photosystems supercomplexes, triggering their destabilization and reorganization in both cyanobacteria and plants; (4) state transitions exhibit at least two-exponential kinetics; (5) the movement of MGDG-PBS complexes together during the progression of State transitions. The PAL mutant-related results clarify the indispensable role of the PBSs in light-quality-changes-induced State transitions and demonstrate their physiological importance in restoring the redox state of the PQ pool by PBS-dependent surface and membrane reorganization processes. The confirmation of the HMM model in cyanobacteria ensures that State transitions represent a pure biophysical, ancient regulatory mechanism predating the biochemical addition of kinase/phosphatase in eukaryotes. The evolutionary conservation of the HMM model makes it a fundamental principle in photosynthetic regulation.

## Figures and Tables

**Table 1:** Molar concentrations of Chl*a* and PC, along with their molar ratio and the *A*_630_/*A*_683_ absorbance ratio of WT and F124A-PetD mutant of *Synechocystis*, derived from whole-cell absorption spectra using formulas from [38]. Values are mean ± SD (n = 11).

Strain	Chl*a* (mM)	PC (mM)	PC/Chl*a* (mol/mol)	*A*_630_/*A*_683_ (PSII + PBS)/PSI
**WT**	5.12 ± 0.36	2.16 ± 0.19	0.42 ± 0.02	0.69 ± 0.04
**PetD-F124A**	4.48 ± 0.19 ***	2.25 ± 0.09	0.50 ± 0.02 ***	0.82 ± 0.03 ***

Asterisks indicate the level of statistically significant difference between the WT and PetD-F124A, with *p* < 0.001 (***) according to a paired *t*-test.

**Table 2:** 77K fluorescence emission ratios of *Synechocystis* WT and PetD-F124A mutant cells, derived from spectra obtained upon 590 nm and 436 nm excitation. The values are mean ± SE from at least three independent experiments.

Strain, State	F662/F650 (λ_exc_ 590) PC to Bulk APC	F685/F662 (λ_exc_ 590) Bulk APC to APC_TE_/CP43	F693/F685 (λ_exc_ 590) APC_TE_ to CP47	F722(λ_exc_ 590)/F722(λ_exc_ 436) PBS to PSI
**WT**				
State 1	1.230 ± 0.006	1.232 ± 0.041	1.207 ± 0.014	0.869 ± 0.005
State 2	1.227 ± 0.009	1.091 ± 0.058	1.192 ± 0.024	0.897 ± 0.006
**PetD-F124A**				
State 1	1.106 ± 0.010 *	1.166 ± 0.011	1.149 ± 0.007 *	1.053 ± 0.003 ***
State 2	1.076 ± 0.016 **	1.133 ± 0.031	1.164 ± 0.023	1.055 ± 0.014 ***

The asterisks indicate the level of statistically significant difference between the respective State 1 and State 2 of WT and PetD-F124A, with *p* < 0.05 (*), *p* < 0.01 (**), and *p* < 0.001 (***) according to a paired *t*-test.

**Table 3:** PAM and State transitions parameters (±SE) for *Synechocystis* WT and PetD-F124A cells.

Species	WT	PetD-F124A
Fo	0.115 ± 0.005	0.137 ± 0.004 *
Fm	0.179 ± 0.006	0.214 ± 0.008 *
Fv	0.064 ± 0.002	0.077 ± 0.005 *
Fv/Fm	0.360 ± 0.006	0.360 ± 0.011
Φ_PSII_ (State1) = (Fm1 − Fi)/Fm1	0.427 ± 0.013	0.379 ± 0.007 *
Φ_PSII_ (State2) = (Fm2 − Fii′)/Fm2	0.370 ± 0.007	0.343 ± 0.004 *
1-qP = (Fi − Fo′)/(Fm2 − Fo)	0.027 ± 0.006	0.051 ± 0.005 *
		
qT = (Fm1 − Fm2)/Fm1, %	8.70 ± 1.33	4.16 ± 0.52 ***
qS = ((Fi’ − Fi) − (Fii’ − Fii))/(Fi’ − Fi)	0.501 ± 0.034	0.282 ± 0.022 ***
t_ind1→2_, s	3.9 ± 0.2	4.2 ± 0.6
t_ind2→1_, s	1.3 ± 0.26	4.0 ± 0.62 *

The asterisks indicate significant differences with the respective parameters of WT, with *p* < 0.05 (*), and *p* < 0.001 (***) according to a paired *t*-test.

**Table 4:** Two exponential fits of fluorescence decrease during the State 1→State 2 transition or increase during the State 2→State 1 transition of fluorescence in WT and PetD-F124A cells. Amplitudes (Ai) and halftimes (t_1/2_i) of exponential fitting during State transitions.

State Transition, Specie (±SE)	A1 (%) ^a^	t_1/2_1 (s)	A2 (%) ^a^	t_1/2_2 (s)	A3 (%) ^a^	t_1/2_3 (s)	∑Ai as % of ∑Ai of st1→st2 in WT ^a^	Amplitude Average t_1/2_*av* (s) ^b^
**State 1→State 2**								
WT (n = 8)	25.7 ± 3.5	7.9 ± 1.6	-	-	74.3 ± 3.5	210 ± 10	100	156 ± 6.5
PetD-F124A (n = 16)	29.8 ± 1.6	4.5 ± 0.5	-	-	29.9 ± 1.6 ***	229 ± 17.5	59.7 ***	113 ± 8 ***
**State 2→State 1**								
WT (n = 4)	15.7 ± 1.6	2.7 ± 0.8	15.9 ± 1.6	51 ± 4	-	-	31.6	25.4 ± 2.1
PetD-F124A (n = 7)	14.1 ± 0.9	7.4 ± 2.1 *	15.8 ± 0.9	78 ± 11 *	-	-	29.9	43.8 ± 5.9 *

^a^ The amplitudes of the two components are normalized relative to the sum of the amplitudes of the State 1→State 2 transition, taken as 100%. ^b^ The amplitude-weighted average halftime is t_1/2_*av* = ΣA*i* t_1/2_*i*/ΣA*i*. The asterisks indicate significant differences with the respective parameter of the WT, with *p* < 0.05 (*), and *p* < 0.001 (***) according to a paired *t*-test (n is indicated in the first column = 5 ± SE).

**Table 5:** Kinetics of State Transitions in *Arabidopsis* Col-0 and *stn7* Leaves.

State Transitions, Specie	A1 (%)	t_1/2_1 (s)	A2 (%)	t_1/2_2 (s)	A3 (%)	t_1/2_3 (s)	Amplitude Average t_1/2_*av* (s) ^a^
**State 1→State 2**							
Col-0 (n = 4)	-	-	69 ± 4	81 ± 8	31 ± 4	195 ± 30 ^b^	114 ± 11
*Stn7* (n = 4)			***	***	***	***	***
**State 2→State 1**							
Col-0 (n = 5)	-	-	-	-	100	502 ± 23 ^c^	
*Stn7* (n = 4)					***	***	

^a^ The amplitude-weighted average halftime is t_1/2_*av* = ΣA*i* t_1/2_*i*/ΣA*i*. ^b^ The fluorescence decrease during the State 1→State 2 transition (Figure 12g) is best fitted by two-exponential decay components: Amplitudes (Ai) in percent and halftimes (t_1/2_i) of the exponential fitting. ^c^ A polynomial or linear function best fitted the fluorescence increase during the State 2→State 1 transition. The halftime corresponds to the point at which the fluorescence increases by 50%. The asterisks indicate significant differences relative to the WT parameter, with *p* < 0.001 (***) by paired *t*-test (n is indicated in the first column, ±SE).

## Data Availability

Data are contained within the article.

## Abbreviations

The following abbreviations are used in this manuscript:

APC	allophycocyanin
APC_TE_	APC terminal emitter
CD	circular dichroism
Chl*a*	chlorophyll *a*
cyt*b*_6_*f*	cytochrome *b*_6_*f* complex
DGDG	digalactosyldiacylglycerol
DSC	differential scanning calorimetry
LHC	light-harvesting complex
MGDG	monogalactosyldiacylglycerol
OD	optical density
PBP	phycobiliprotein
PBS	phycobilisome
PC	phycocyanin
PG	phosphatidylglycerol
PQ	plastoquinone
PSI	photosystem I
PSII	photosystem II
SQDG	sulfoquinovosyldiacylglycerol
WT	wild type
